# Life-threatening multiorgan immune-related toxicities complicated by sepsis after anti-PD-1 therapy with complete tumor regression: a case report and literature review

**DOI:** 10.3389/fimmu.2026.1830699

**Published:** 2026-07-01

**Authors:** Jun Dong, Lijun Zhang, Lei Yu, Jun Wang, Jijie Huang, Xiaoyan Wu, Ping-An Wu, Suling Chen, Xixi Chen, Wenjuan Zhu, Yuan Qu, Jishi Li, Victor Ho Fun Lee

**Affiliations:** 1 Clinical Oncology Center, The University of Hong Kong-Shenzhen Hospital, Shenzhen, China; 2 Division of Rheumatology, Department of Medicine, The University of Hong Kong-Shenzhen Hospital, Shenzhen, China; 3 Department of Intensive Care Unit, The University of Hong Kong-Shenzhen Hospital, Shenzhen, Guangdong, China; 4 Department of Hematology, The University of Hong Kong-Shenzhen Hospital, Shenzhen, Guangdong, China; 5 Department of Dermatology, The University of Hong Kong-Shenzhen Hospital, Shenzhen, China; 6 Department of Surgery, The University of Hong Kong-Shenzhen Hospital, Shenzhen, China; 7 Division of Nephrology, Department of Medicine, University of Hong Kong-Shenzhen Hospital, Shenzhen, China; 8 Department of Clinical Oncology, School of Clinical Medicine, Li Ka Shing Faculty of Medicine, The University of Hong Kong, Hong Kong, Hong Kong SAR, China

**Keywords:** head and neck cancer, immune-related adverse events, immunotherapy, intravenous immunoglobulin, multiorgan failures

## Abstract

**Background:**

Immune checkpoint inhibitors (ICIs) enhance antitumor immunity but can disrupt immune tolerance, leading to immune-related adverse events (irAEs) affecting multiple organs. Simultaneous life-threatening multiorgan irAEs remain rare and poorly characterized, particularly in head and neck cancer.

**Case presentation:**

A 73-year-old man with sinonasal squamous cell carcinoma developed fulminant multisystem immune toxicity after a single dose of pembrolizumab combined with nab-paclitaxel. The clinical course was marked by Stevens–Johnson syndrome, hematologic and renal failure, pneumonitis, metabolic dysregulation, and recurrent sepsis requiring intensive care. A multidisciplinary strategy, including team discussion, intensive care, corticosteroids and two courses of intravenous immunoglobulin (IVIG), was employed. Dose-escalated corticosteroids may cause potent immunosuppression and potentially raise the risk of opportunistic infections. IVIG serves as an immunomodulator which can balance immune control with infection risk. His organ function recovered despite concurrent catheter-related systemic infection. Remarkably, the patient achieved complete tumor regression after one treatment cycle and disease-free duration was 6 months.

**Conclusion:**

This case illustrates the extreme spectrum of immunologic dysregulation associated with PD-1 blockade and highlights the importance of rapid and early diagnosis, multidisciplinary management, and risk-adapted immunomodulation. IVIG may represent a pragmatic therapeutic strategy when escalation of immunosuppression is limited by infection risk. Further investigation is needed to optimize prediction and management of life-threatening multiorgan irAEs.

## Background

The blockade of the PD-1/PD-L1 axis is a widely used therapeutic modality in the treatment of head and neck squamous cell carcinoma (HNSCC) (1). However, immune checkpoint inhibitors (ICIs) can also disrupt immune tolerance and give rise to immune-related adverse events (irAEs), which may affect virtually any organ system. Most irAEs are low grade and manageable, while severe and life-threatening toxicities remain a major clinical challenge. It has been reported that the incidence of irAEs induced by ICIs was 40.0% for any grade and 19.7% for high grade (2). The side effects may involve any organ system with a median onset of approximately 40 days (3). Multiorgan irAEs are uncommon and are typically reported as sequential or limited-severity events. The incidence of severe irAEs ranged from 5.4% to 19.7%, whereas multisystem irAEs occurred in 0.34% to 17% of patients. Notably, concurrent-onset irAEs involving multiple organs were reported in less than 1% of cases (2, 4–9). In particular, simultaneous, life-threatening involvement of multiple organ systems is exceedingly rare and poorly characterized, particularly in patients with head and neck cancer.

The mechanism underlying anti-PD-1 therapy induced irAE remains poorly understood. The prevailing hypothesis suggests that inhibition of PD-1 activates pre-existing tissue-resident memory T cells, leading to an attack on normal tissues, and promotes high level of cytokine secretion (10). In principle, the management of irAEs from ICIs primarily relies on immunosuppressive therapy (3, 11, 12). Systemic corticosteroids remain the fundamental treatment for irAEs. The dilemma is that prolonged or intensified immunosuppression may increase the risk of infection and promote tumor progression. According to ASCO and ESMO guidelines, intravenous immunoglobulin (IVIG) has emerged as a potential immunomodulatory strategy in selected cases, particularly when conventional immunosuppressive escalation is contraindicated or poorly tolerated (3, 11). Management of multiple irAE poses substantial challenges to oncologists which usually necessitates a multidisciplinary team (MDT) management approach. Here, we present a rare case in which a 73-year-old male developed potentially fatal multi-organ failure complicated by severe infection following a single dose of anti–PD-1 therapy combined with nab-paclitaxel for his advanced sinonasal squamous cell carcinoma. The patient recovered from these life-threatening complications and reached a complete response (CR) through multidisciplinary management incorporating corticosteroids and IVIG. This case highlights the diagnostic and therapeutic challenges and provides practical insights into risk-balanced immunomodulatory management in this emerging clinical scenario.

## Case presentation

**Figure 1:**
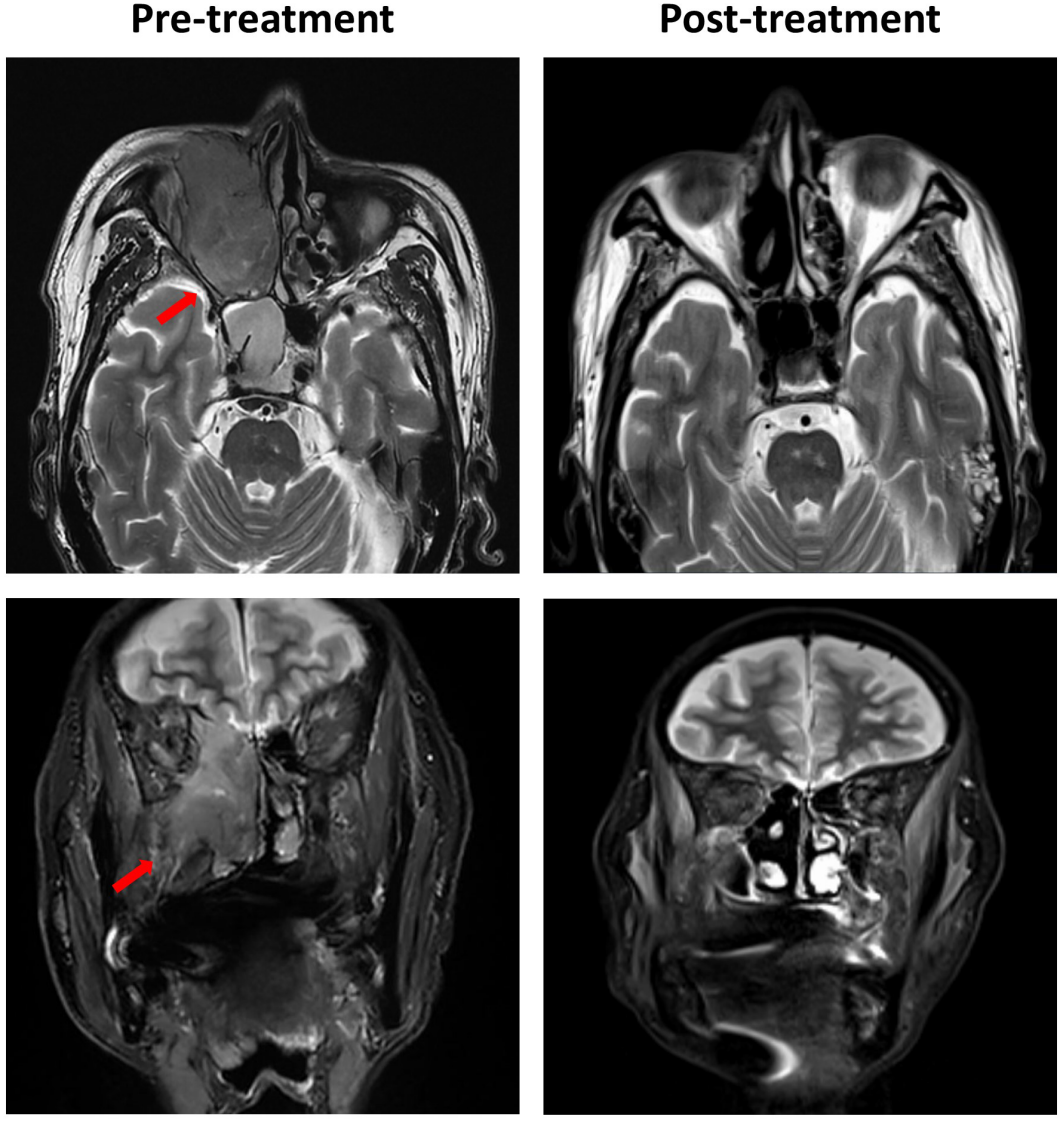
Nasopharynx MRI with contrast revealed tumor invasion pre and post immunotherapy and chemotherapy.

A 73-year-old man with a history of lung adenocarcinoma (pT1bN0M0), chronic obstructive pulmonary disease (COPD), and atopic dermatitis presented with right-sided epistaxis and progressive visual impairment. Magnetic resonance imaging (MRI) and positron emission tomography/computed tomography (PET/CT) revealed an extensive lesion involving the right nasal cavity, maxillary sinus, and ethmoid sinus, with invasion of adjacent structures (**Figure 1**). Biopsy of the nasal cavity lesion demonstrated moderately differentiated squamous cell carcinoma with high PD-L1 expression [PD-L1 (22C3) combined positive score (CPS) 85, tumor proportion score (TPS) 70].

Given the patient’s rapidly progressive tumor-related symptoms, including severe pain and progressive orbital swelling with proptosis, the MDT recommended first-line treatment with pembrolizumab in combination with nab-paclitaxel. This strategy balanced anticipated response efficacy with chemotherapy-related toxicities.

**Figure 2:**
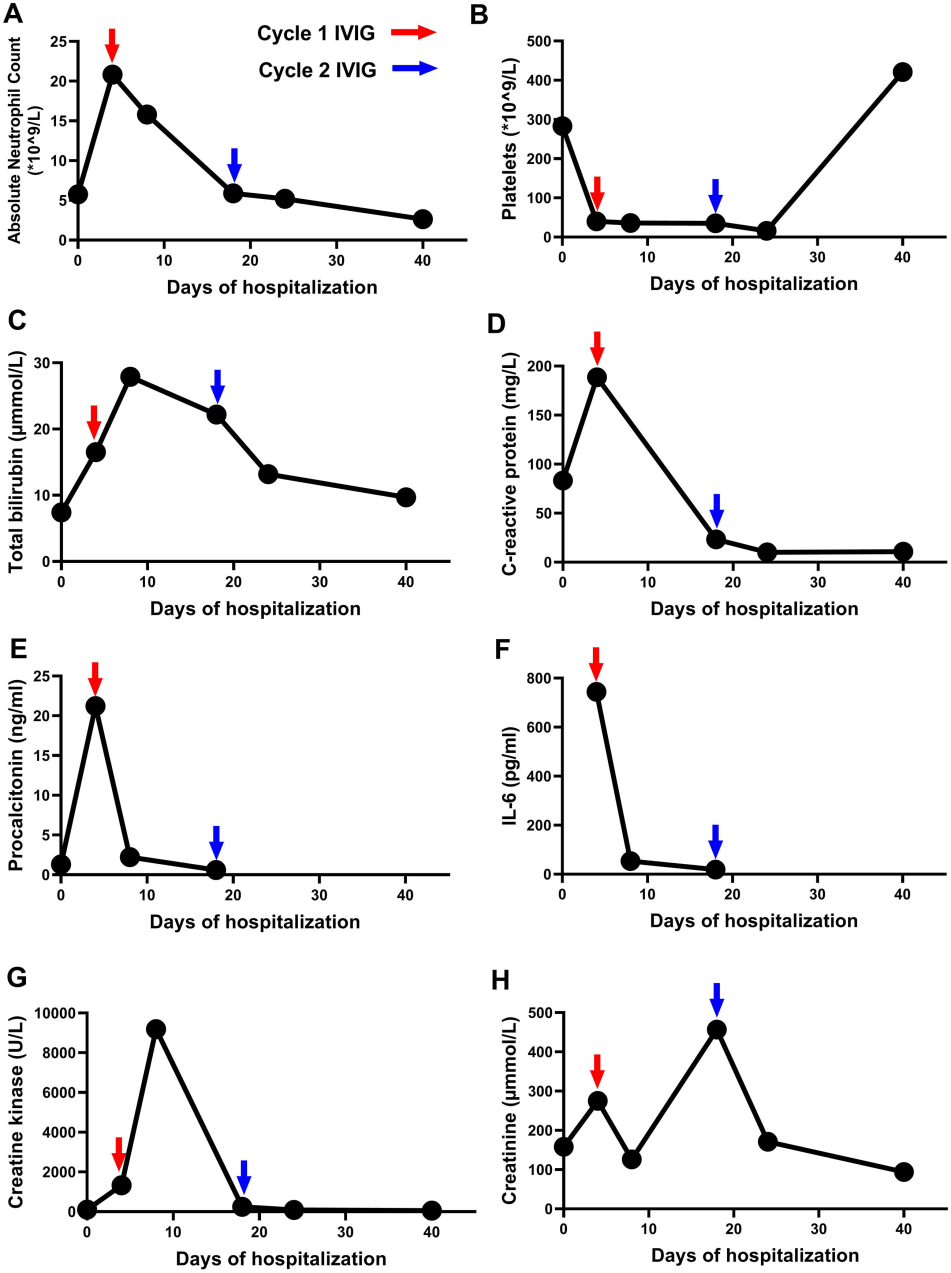
The trend of various laboratory parameters including absolute neutrophil count **(A)**, platelets **(B)**, total bilirubin **(C)**, C-reactive protein **(D)**, procalcitonin **(E)**, IL-6 **(F)**, creatine kinase **(G)** and creatinine **(H)**. The arrows denoted time points for the initiation of intravenous immunoglobulin (IVIG).

Eight days post-pembrolizumab and nab-paclitaxel, he reported marked symptomatic improvement, accompanied by an improvement in Eastern Cooperative Oncology Group (ECOG) performance status from 2 to 1; however, no radiologic examination was performed because the time point for evaluation had not yet been reached. Dermatology consultation was obtained at 12 days post-systemic therapy for progressively worsening pruritic skin eruptions, for which the clinical diagnosis was most consistent with either atopic dermatitis or a grade 2 ICI-related cutaneous adverse event. Seventeen days after chemo-immunotherapy, he complained of fever and chills, and was admitted to our hospital via the emergency department. On admission, his vital signs revealed a temperature of 38.1 °C, blood pressure of 112/66 mmHg, heart rate of 102 beats per minute, and respiratory rate of 18 breaths per minute. Physical examination identified grade 2 diffuse and generalized rash. His laboratory findings are summarized in **Table 1** and **Figure 2**. In the context of markedly elevated inflammatory markers, active infection was suspected. Empiric broad-spectrum antibiotic therapy with piperacillin–tazobactam was initiated.

**Table 1:** Laboratory values on admission, after intravenous immunoglobulin and at the time of hospital discharge.

Laboratory values	Reference value	On admission	Initiation of cycle 1 IVIG (day 4)	End of cycle 1 IVIG (day 8)	Initiation of cycle 2 IVIG (day 18)	2 days post-cycle 2 IVIG (day 24)	3 days before hospital discharge (day 40)
WBC (x10^9/L)	3.5-9.5	7.21	21.81	19.39	6.28	6.02	3.37
ANC (x10^9/L)	1.8-6.3	5.77	20.83	15.80	5.89	5.19	2.64
Hemoglobin (g/L)	130-175	115	82	65	83	63	73
Platelet count (x10^9/L)	125-350	283	40	36	35	16	421
Total bilirubin (µmmol/L)	0-23	7.4	16.5	27.9	22.2	13.2	9.7
AST (U/L)	15-40	41	107	322	37	63	46
ALT (U/L)	9-50	18	25	171	41	54	51
ALP (U/L)	45-125	76	59	123	118	166	219
CRP (mg/L)	<5	83.2	188.5	NA	23.2	10.1	10.7
PCT (ng/ml)	<0.1	1.31	21.21	2.22	0.59	NA	NA
IL-6 (pg/ml)	<7	NA	744.5	53.6	19	NA	NA
LDH (U/L)	120-250	418	1281	NA	602	260	281
CK (U/L)	<190	105	1329	9187	249	84	51
Creatinine (µmol/L)	57-111	158	275	126	457	171	94
APTT	26.0-40.0	59.2*	96.6	41.3	27.5	15.4	NA
PT-INR	0.8-1.20	2.35*	2.17	1.15	1.46	1.24	NA
Serum potassium (mmol/L)**	3.5-4.9	NA	4.5	4.1	NA	NA	NA
PH**	7.35-7.45	NA	7.305	7.389	NA	NA	NA
pCO_2_ (kPa)**	4.67-6.40	NA	6.01	4.26	NA	NA	NA
pO_2_ (kPa)**	11.1-14.4	NA	13.7	8.3	NA	NA	NA
HCO_3_^-^ (mmol/L)**	22-26	NA	22.3	19.2	NA	NA	NA
base excess (mmol/L)**	-2-3	NA	-4	-6	NA	NA	NA

*the sample was collected two days after admission; **blood gas parameters (pH, pCO_2_, pO_2_, HCO_3_^-^, base excess) were obtained from arterial blood samples.

NA, not available.

As the patient developed hemodynamic instability with progressive deterioration in vital signs, he was transferred to the intensive care unit (ICU) after two days in the general ward. At the same time, Stevens–Johnson syndrome (SJS; **Supplementary Figure 1**), severe myalgia, and acute renal failure became clinically apparent. Approximately 40% body surface area was covered by skin erythema and sloughing, accompanied by epidermal detachment and genital mucosal detachment. Aggressive supportive managements including significant volume resuscitation, vasopressor support, continuous renal replacement therapy (CRRT), and escalation to broad-spectrum antimicrobial therapy (meropenem) were promptly initiated. Computed tomography (CT) demonstrated acute cholecystitis with dilation of the common hepatic duct and wall thickening of the common bile duct, for which emergent cholecystostomy was performed.

Based on MDT discussions involving oncologists, intensivists, infectious disease specialists, nephrologists, rheumatologists, and dermatologists, ICI-related multiorgan injuries was diagnosed, with acute cholecystitis considered a contributing factor to the severe infectious process. Given the presence of multiorgan dysfunction affecting the dermatologic, muscular, hematologic, gastrointestinal, renal, and respiratory systems, intravenous methylprednisolone (1 mg/kg twice daily) and esomeprazole were given. Two days after initiating corticosteroids, intravenous immunoglobulin (IVIG) was administered at a total dose of 2 g/kg divided over 5 days due to continuously increasing infectious markers (**Figures 2A, D–F**) and low platelet count of 40 × 10^9^/L (**Table 1**). Within 48 hours of combined corticosteroid and IVIG therapy, the patient’s vital signs showed a trend toward stabilization, inflammatory markers declined and renal function was recovering. And then the patient was successfully weaned from vasopressors and transferred to general ward. It supported the diagnosis of irAEs and demonstrating rapid responsiveness to immunomodulatory treatment.

During corticosteroid tapering, although most organ toxicities improved, grade 4 thrombocytopenia (platelet of 35 × 10^9^/L) and renal dysfunction (creatinine of 457 µmol/L) persisted (**Table 1**). Platelet transfusion and recombinant human thrombopoietin were administered to facilitate platelet recovery. The patient also experienced episodes of transient hypoxemia and intermittent melena during this recovery phase. A second MDT discussion was convened to evaluate the possibility of steroid-refractory immune-related hematologic toxicity and the potential need for second-line immunosuppressive agents. Given the recent history of severe infection and the presence of chronic gastrointestinal bleeding without significant hemoglobin decline, and after additional evaluation to exclude alternative causes of thrombocytopenia, consensus was reached to administer a second course of IVIG rather than escalate corticosteroid therapy or introduce additional immunosuppressive agents, in order to minimize the risk of secondary or recurrent infection. Following the second IVIG course (the same dose as the first course), platelet counts gradually recovered, and serum creatinine levels declined in parallel.

On day 30, the patient developed a second episode of sepsis, with blood cultures positive for *Klebsiella pneumoniae*, which was considered most consistent with a central venous catheter (CVC)-related bloodstream infection. This episode was promptly recognized and successfully managed with targeted antibiotic therapy and the CVC was removed immediately, without further clinical deterioration. Intravenous methylprednisolone at a dose of 30 mg daily was maintained for a further 10 days following the second septic episode, during which inflammatory markers, hematologic parameters, and renal function continued to show sustained improvement. Corticosteroid therapy was maintained until vital sign became stable and was subsequently transitioned to oral methylprednisolone followed by gradual tapering over 5 weeks.

**Figure 3:**
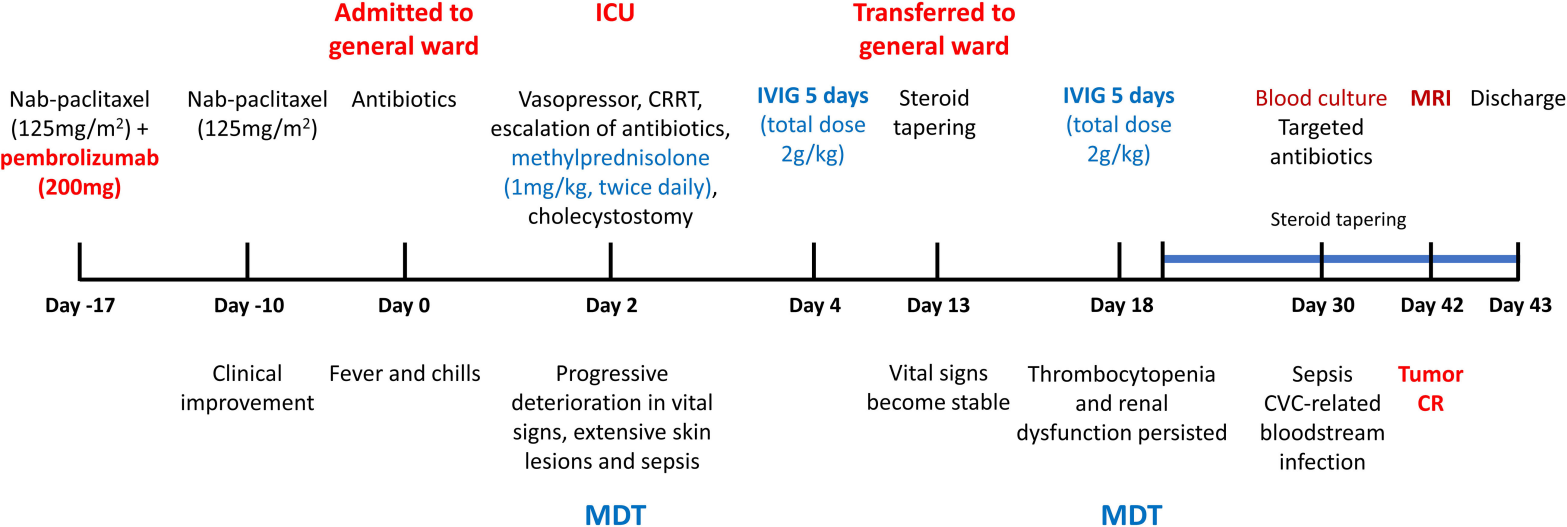
Clinical course of the diagnosis and treatment. CRRT, continuous renal replacement therapy; IVIG, intravenous immunoglobulin; CVC, central venous catheter; CR, complete response.

Notably, MRI scan was performed on day 42 and demonstrated CR (**Figure 1**). Unfortunately, the tumor recurred 6 months after the initial evaluation. Chemotherapy and cetuximab were then administered. Considering the potential risk of irAEs, immunotherapy was not re-challenged. The patient was discharged on day 43 of hospitalization. Clinical course of the diagnosis and treatment was summarized in **Figure 3**.

## Differential diagnosis

The differential diagnoses were broad (summarized in **Supplementary Table 1**) and included sepsis as a potential contributor to multiorgan failure, particularly given the acute cholecystitis identified after admission. However, blood cultures obtained on day 0 and 2 were negative, serum bilirubin level was only mildly elevated, and severe cutaneous and muscular manifestations are uncommon in sepsis alone. Taken together, these findings suggested that infection alone was unlikely to fully account for the extent and pattern of multiorgan dysfunction.

ADAMTS13 activity, ADAMTS13 IgG, and ADAMTS13 inhibitor assays were performed to evaluate thrombotic thrombocytopenic purpura (TTP), and all results were negative. Bone marrow biopsy was considered but was not performed due to severe thrombocytopenia. In light of the laboratory workup including the temporal trends of inflammatory and infectious markers (**Figures 2D–F**), as well as negative TTP-related assays, the thrombocytopenia was considered most consistent with ICI-related hematologic toxicity. The acute kidney injury was primarily attributed to immunotherapy, as renal function continued to deteriorate despite adequate control of infection (**Figures 2D–F, H**).

The etiology of hypoxemia was considered multifactorial and included immune-related pneumonitis, sepsis-associated respiratory dysfunction, and underlying COPD. Targeted next-generation sequencing (tNGS) of bronchoalveolar lavage fluid (BALF) was negative for infectious pathogens, and chest CT demonstrated interstitial lung changes of the lung parenchyma, supporting a diagnosis of immune-related pneumonitis.

In the absence of clinical, endoscopic, or radiologic evidence of immune-mediated colitis - and notably without accompanying diarrhea - the gastrointestinal bleeding was considered unlikely to be immune-related. It was most likely attributed to corticosteroid-associated mucosal injury supported by stable hemoglobin level (**Table 1**), with thrombocytopenia regarded as a possible but less likely contributing factor.

## Treatment

MDT collaboration enabled rapid recognition of disease severity and early initiation of intensive supportive care, including broad-spectrum antimicrobial therapy, vasopressor support, CRRT, and systemic corticosteroids. Methylprednisolone was initiated at 1 mg/kg twice daily and then tapered once the disease stabilized, with a reduction of 10 mg every 3–7 days over 5 weeks. These interventions were critical in preventing further clinical deterioration induced by irAEs and concurrent infection. IVIG (2 g/kg over 5 days) was incorporated as an immunomodulatory strategy to mitigate the cytokine-mediated inflammatory response associated with irAEs and sepsis, while avoiding escalation to more potent immunosuppressive agents that could increase the risk of secondary or recurrent infection. Given the achievement of CR, there was no indication to re-challenge immunotherapy or chemotherapy. The disease−free duration was 6 months. After recurrence, paclitaxel−based chemotherapy and cetuximab were administered. Given the life−threatening ICI−related toxicities, immunotherapy was not rechallenged.

## Literature review

A systematic search of PubMed was conducted to identify published case reports describing ICI-related multiorgan toxicities between January 2000 through January 2026. The search strategy included keywords such as immunotherapy, immune-related adverse events, multiorgan, and cancer, along with their relevant synonyms and variations. A total of 150 articles were identified, of which 31 case reports describing 35 individual patients met the inclusion criteria.

A summary of the identified cases is shown in **Supplementary Table 2**, and the distribution of affected organ systems is illustrated in **Supplementary Figure 2**. Hepatotoxicity was the most frequently reported irAE, followed by renal toxicity. In addition to systemic corticosteroids, second-line immunosuppressive agents-including infliximab, mycophenolate mofetil, and tocilizumab-were commonly utilized in patients with multiple irAEs.

Among the 35 reported patients, 12 (34.3%) experienced involvement of more than three organ systems with severe immune-related toxicities. Systemic corticosteroids were administered in all 12 cases, and intravenous immunoglobulin (IVIG) was used in 4 of these patients (33%). These findings suggest that immunosuppression remains the cornerstone of management for severe irAEs, while sustained immunosuppression may potentially increase the risks of infection and dampen antitumor immunity. Notably, majority of these patients were not treated with antibiotics (**Supplementary Table 2**).

IVIG serves as an immunomodulator by attenuating pathogenic immune responses without inducing profound immunosuppression. Nevertheless, given the limited number of reported cases and the high heterogeneity in clinical presentations and treatment approaches, these observations should be interpreted with caution. Further prospective studies are merited to define optimal management strategies for patients with life-threatening multiorgan irAEs.

## Discussion

The reported incidence of multiorgan irAEs ranges from 5.4% to 60% and majority of them are G1–2 toxicities (7, 8, 13, 14). In contrast, life-threatening multiorgan irAEs remain exceedingly rare, and published data guiding their diagnosis and management are limited. In the present case, the patient developed four concurrent grade 4 irAEs after a single dose of pembrolizumab, representing an exceptionally rare clinical scenario. To the best of our knowledge, no prior case of simultaneous life-threatening multiorgan irAEs in HNSCC has been published. Notably, despite the severity of toxicities, the patient recovered and achieved a complete tumor response. Our experience may provide valuable insights into management in patients with head and neck cancer who suffering from immunotherapy-induced multiorgan failure.

Key factors contributing to successful management of this case included (1): early recognition and diagnosis of irAEs (2), timely initiation of corticosteroids with adjuvant immunomodulatory treatment using IVIG, and (3) close MDT collaboration. Diagnosing multiorgan irAEs is inherently challenging because clinical manifestations may be severe, heterogeneous, and temporally variable, and irAEs are frequently diagnoses of exclusion. Accordingly, MDT involvement is essential to establish an accurate diagnosis and implement appropriate management. Naidoo et al. (15) have demonstrated that virtual MDT collaboration can facilitate earlier identification and improved management of irAEs. In published series, hepatic toxicity is among the most frequently reported irAEs (**Supplementary Figure 2**), and distinguishing immune-associated injury from alternative etiologies such as chemotherapy toxicity or infection is critical when determining whether to apply immunosuppression. In this case, rapid clinical deterioration with sequential dermatologic, renal, hematologic, and muscular involvement prompted two MDT discussions that were pivotal in refining the diagnosis and guiding treatment decisions.

Timely administration of systemic corticosteroid is essential to reduce the risk of irreversible organ injury and fatal outcomes. Conversely, when concomitant infection is suspected or confirmed, escalation of immunosuppression must be weighed carefully. Immunomodulator therapy may be considered as a substitute intervention in the scenario. IVIG is one of the most commonly used immunomodulator, which can neutralize pathogenic autoantibodies, impair cytokine-mediated inflammatory cascades, and regulate T-cell responses toward a less pro-inflammatory phenotype. The recommended dosage is 2 g/kg over 2–5 days in divided doses of 400–500 mg/kg per ASCO guideline (3). In this case, consensus was not reached to escalate to second-line immunosuppressive agents, the application of IVIG was supported when hematologic and renal toxicities persisted despite prolonged corticosteroid therapy. Two courses of IVIG were associated with dramatic clinical and laboratory improvement, suggesting its potential role as a pragmatic option for selected patients with severe multisystem irAEs, particularly when infection risk limits immunosuppressive escalation.

According to ASCO and ESMO guideline (3, 11), the indications of IVIG include hematologic irAEs, severe cutaneous adverse reaction, pneumonitis, myositis, myasthenia gravis, Guillain-Barre´ syndrome, encephalitis, demyelinating disease and uveitis. IVIG is usually utilized as a second-line therapy when patients are refractory to steroid treatment. Ruf et al. reported that profiles of second line therapies of irAEs and IVIG was widely used in cutaneous toxicities, myositis and neurological toxicities (16). Pu et al. also reported that 38.1% patients with ICI-induced SJS received systemic corticosteroids combined with IVIG (17). In addition, IVIG was one of most used second line immune modulators in patients with melanoma who presented with irAEs (18).

Notably, during the recovery phase, the patient developed a second episode of sepsis that was clinically consistent with a CVC-related bloodstream infection and was promptly controlled with targeted antibiotic therapy. This observation underscores the vulnerability of patients with severe multiorgan immune-related toxicities to iatrogenic infectious complications and further supports a cautious, risk-adapted immunomodulatory strategy rather than aggressive escalation of immunosuppressive therapy.

Accumulating evidence suggests that dermatologic irAEs following ICI therapy indicate robust immune activation and are associated with improved overall survival (OS) (14, 19, 20). Patients with cutaneous toxicity appear to have a substantially increased risk of multisystem irAEs (21), and multiple studies have shown that multisystem toxicities correlate with prolonged progression-free survival and OS compared with single-organ irAEs (7, 8, 13, 14, 22). However, the association does not prove causality in a single case, and severe irAEs may also increase mortality. In the present case, the onset of cutaneous toxicity was earliest, underscoring the need for heightened vigilance for subsequent multisystem involvement. Consistent with the onset of cutaneous toxicity was earliest, a CR was achieved after a single cycle of chemo-immunotherapy. Interestingly, Wan et al. have reported a co-occurrence patterns of irAEs in which cutaneous, musculoskeletal, and renal irAEs tended to co-occur with all other organ systems (14), which was concordant with this patient’s clinical course.

Published reports suggest that multisystem irAEs occur more frequently in male patients and in immunogenic tumor types such as melanoma and lung cancer (**Supplementary Table 2**). In this case, the patient demonstrated high PD-L1 expression, suggestive of active immune profile referred to as the “hot” (immune-inflamed) phenotype, and had a history of autoimmune disease (atopic dermatitis), both of which may have predisposed him to exaggerated immune activation and multisystem toxicity.

Importantly, severe irAEs developed 17 days after the first infusion of anti-PD-1 therapy. A prospective study reported that 1.96% of patients experienced irAEs after a single ICI dose, with onset within 21 days and grade 3–5 toxicities occurring in 32.8% of cases (23). These findings emphasize the need for close clinical and laboratory monitoring closely after treatment initiation and heightened awareness of the potential for rapid-onset multisystem irAEs. At present, no validated biomarkers reliably predict patients at high risk for fatal or multisystem toxicities, underscoring the urgent need for further research.

It is also important to acknowledge that the patient received combination therapy with nab-paclitaxel and pembrolizumab, and these agents may exert synergistic antitumor effects. Taxane-based chemotherapy can enhance tumor antigen release, facilitate the differentiation of myeloid-derived suppressor cells into dendritic cells and modulate the tumor microenvironment (24), thereby potentially augmenting the activity of ICI. Such synergistic effect may have contributed to the rapid symptomatic improvement and complete tumor response observed after a single treatment cycle. Guiard et al. reported that prior anti-PD-1 exposure was associated with a significantly better response to taxane monotherapy in patients with recurrent and metastatic HNSCC after platinum failure (25). A possible explanation is that taxanes exhibit an immunomodulatory effect that could act synergistically with residual prolonged anti-PD-1 activity. Nevertheless, the pattern, severity, and multisystem nature of the observed toxicities strongly support a predominantly immune-mediated etiology rather than chemotherapy-related toxicity. He et al. summarized 12 clinical trials and found that hematologic AEs were most frequently reported, followed by gastrointestinal toxicities and then rash events (26). Neal et al. reported that toxic erythema was most common skin toxicity induced by taxanes with an incidence of 17.6% (27). Nab-paclitaxel is not typically associated with acute, simultaneous involvement of multiple organ systems, nor with severe cutaneous manifestations such as SJS, immune-mediated myositis, immune-related hematologic and renal failure. In contrast, the clustering of dermatologic, musculoskeletal, renal, hematologic, and pulmonary toxicities; the rapid onset after treatment initiation; and the prompt responsiveness to corticosteroids and IVIG are all characteristic of ICI-related adverse events. Taken together, these features indicate that the life-threatening multisystem toxicities in this patient were most consistent with immune dysregulation induced by anti-PD-1 therapy rather than chemotherapy.

In conclusion, this case highlights both the transformative efficacy and the potential for severe toxicity associated with ICIs. It underscores the importance of early recognition, MDT-driven decision-making, and individualized immunomodulatory strategies in the management of life-threatening multiorgan irAEs. As immunotherapy continues to reshape the oncologic treatment landscape, further clinical and translational studies are needed to improve risk stratification and optimize management for these rare but devastating complications.

## Data Availability

The original contributions presented in the study are included in the article/**Supplementary Material**. Further inquiries can be directed to the corresponding authors.
