# Supplementary material for: Life-threatening multiorgan immune-related toxicities complicated by sepsis after anti-PD-1 therapy with complete tumor regression: a case report and literature review
Source: Front Immunol. 2026 Jul 1;17:1830699. doi: 10.3389/fimmu.2026.1830699 (PMC13369593; doi:10.3389/fimmu.2026.1830699)
Supplement: Supplementary file 1 [file DataSheet1.pdf]

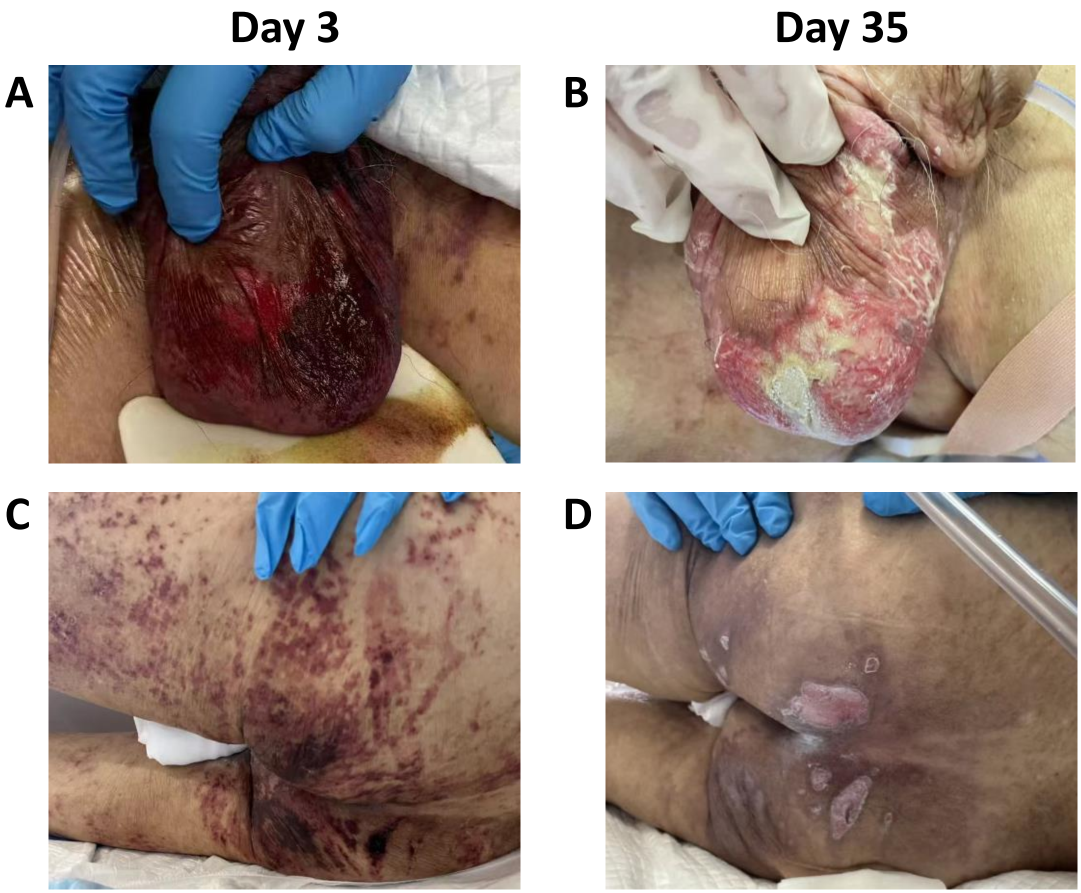

Supplementary figure 1. Skin lesions in scrotum and buttocks. Skin necrosis and detachment of the epidermis were observed in scrotum on day 3 after admission (A) and cutaneous lesions were almost healed after treatments (B). Atypical targetoid lesions, necrosis and detachment of epidermis were found in buttocks (C) and recovered after multiple inventions (D).
