# Supplementary material for: Life-threatening multiorgan immune-related toxicities complicated by sepsis after anti-PD-1 therapy with complete tumor regression: a case report and literature review
Source: Front Immunol. 2026 Jul 1;17:1830699. doi: 10.3389/fimmu.2026.1830699 (PMC13369593; doi:10.3389/fimmu.2026.1830699)
Supplement: Supplementary file 2 [file DataSheet2.pdf]

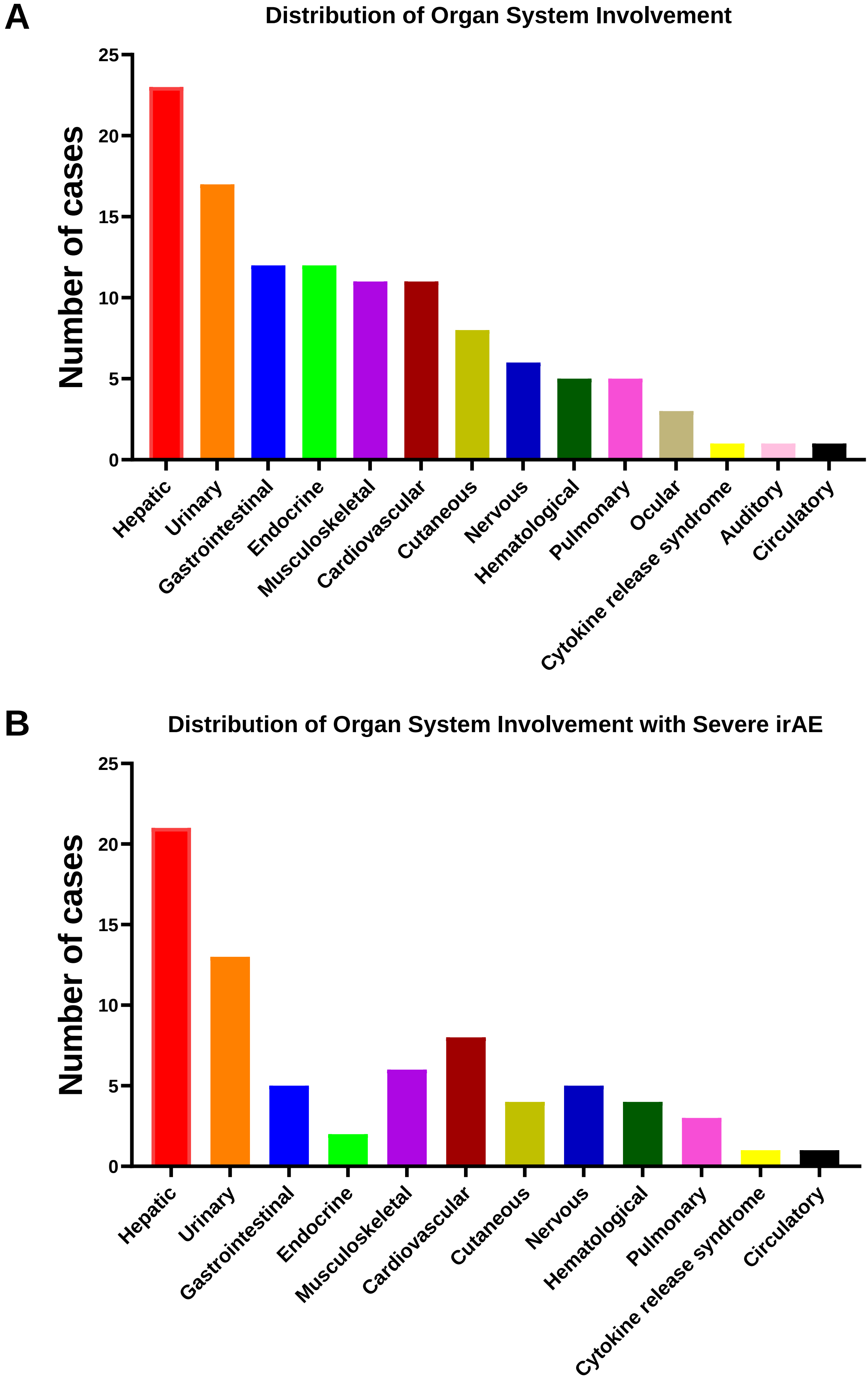

Supplementary figure 2. Distribution of organ system involvement in published case reports of immune related-multi-organ injuries
