# Supplementary material for: Life-threatening multiorgan immune-related toxicities complicated by sepsis after anti-PD-1 therapy with complete tumor regression: a case report and literature review
Source: Front Immunol. 2026 Jul 1;17:1830699. doi: 10.3389/fimmu.2026.1830699 (PMC13369593; doi:10.3389/fimmu.2026.1830699)
Supplement: Supplementary file 3 [file Table1.docx]

**Supplementary table 1. Summary of ICI-related toxicities and differential diagnosis**

|  | Toxicity scale | Symptom and/or lab test results | Differential diagnosis | Etiology |
| --- | --- | --- | --- | --- |
| Severe cutaneous adverse reaction | G4 | Extensive skin lesions | Autoimmune disease | Complication of ICI therapy |
| Myositis | G3-4 | Muscle pain with limiting self-care activity of daily living, CK 9187 U/L | Infection-related | Complication of ICI therapy |
| Immune thrombocytopenia | G4 | Platelet 16*10^9/L | Acquired TTP | Likely attributable to ICI therapy |
| Kidney | G4 | Oliguria, creatinine 457 µmol/L | Sepsis | Likely attributable to ICI therapy |
| Lung | G1 | Hypoxemia | COPD, sepsis-related respiratory dysfunction | Multiple factors including sepsis-related injury, COPD and ICI therapy |
| Gastrointestinal bleeding | NA | Melena | ICI-related colitis | Most likely secondary to thrombocytopenia and maybe attributable to ICI therapy |
