# Supplementary material for: Life-threatening multiorgan immune-related toxicities complicated by sepsis after anti-PD-1 therapy with complete tumor regression: a case report and literature review
Source: Front Immunol. 2026 Jul 1;17:1830699. doi: 10.3389/fimmu.2026.1830699 (PMC13369593; doi:10.3389/fimmu.2026.1830699)
Supplement: Supplementary file 4 [file Table2.docx]

**Supplementary table 2. Summary of published case reports of immune-related adverse events involving multiple systems.**

| No. | Sex | Age  (year-old) | Cancer | Immunotherapy | Toxicities involving systems | Severe toxicities | Treatment | Usage of antibiotics | Clinical outcome |
| --- | --- | --- | --- | --- | --- | --- | --- | --- | --- |
| 1(1) | M | 85 | Melanoma | Nivolumab | Muscular, endocrine, ocular, cardiovascular and hepatic | NA | Steroid | No | Died |
| 2(2) | M | 63 | Melanoma | Ipilimumab and nivolumab | Cutaneous and urinary | Cutaneous and urinary | Steroid and cyclosporine | No | Died |
| 3(3) | F | 60 | Melanoma | Ipilimumab and nivolumab | Ocular, gastrointestinal and cutaneous | Gastrointestinal | Steroid followed by infliximab and vedolizumab | No | Died of tumor progression |
| 4(4) | M | 70 | Melanoma | Pembrolizumab | Cutaneous, urinary, hepatic and nervous | Urinary, hepatic and nervous | Steroid, mycophenolate and IVIG | No | Alive |
| 5(5) | F | 45 | Thymoma | Pembrolizumab | Cardiovascular, muscular and hepatic | Cardiovascular and hepatic | Steroid | No | Died |
| 6(6) | M | 47 | Melanoma | Nivolumab followed by ipilimumab, toxicities occurred during ipilimumab treatment | Musculoskeletal and nervous | Nervous | Steroid | No | Alive |
| 7(7) | F | 64 | Melanoma | Ipilimumab | Ocular and auditory | NA | Steroid | No | Alive |
| 8(8) | F | 52 | Melanoma | Ipilimumab and nivolumab | Hepatic, pancreatic and gastrointestinal | Hepatic | Steroid, mycophenolate mofetil, tacrolimus, sirolimus | Piperacillin  /tazobactam | Alive |
| 9(9) | M | 55 | Lung adenocarcinoma | Pembrolizumab | Cytokine release syndrome, urinary and nervous | Cytokine release syndrome, urinary and nervous | Steroid and tocilizumab | Yes | Alive |
| 10(10) | M | 57 | Lung adenocarcinoma | Nivolumab | Endocrine, pulmonary, cutaneous and hepatic | Cutaneous and hepatic | Steroid and mycophenolate mofetil | No | Alive |
| 11(11) | M | 67 | Lung large-cell neuroendocrine carcinomas | Pembrolizumab | Hepatic, musculoskeletal, cardiovascular and pulmonary | Hepatic, musculoskeletal and cardiovascular | Steroid | Ganciclovir  /cefmetazole | Alive |
| 12(12) | M | 86 | Gastric cancer | Pembrolizumab | Hepatic and pulmonary | Hepatic and pulmonary | Steroid and IVIG | Cefoperazone  Sodium and  Sulbactam  sodium | Alive |
| 13(13) | F | 66 | Colorectal cancer | Sintilimab | Hepatic, musculoskeletal, cardiovascular and cutaneous | Hepatic, musculoskeletal, cardiovascular and cutaneous | Steroid and IVIG | No | Alive |
| 14(14) | M | 55 | Esophageal squamous cell carcinoma | Ipilimumab and nivolumab | Endocrine and hepatic | No severe toxicities | Steroid | No | Alive |
| 15(15) | M | 63 | Hepatocellular carcinoma | Atezolizumab | Endocrine and musculoskeletal | Endocrine | Hydrocortisone | No | Alive |
| 16(16) | M | 67 | Lung adenocarcinoma | Sintilimab | Cardiovascular, muscular and hematological | Cardiovascular and hematological | Steroid | No | Alive |
| 17(17) | M | 60 | Melanoma | Pembrolizumab followed by ipilimumab and nivolumab | Endocrine (pembrolizumab), hematological (dual IO), urinary (dual IO), cardiovascular (dual IO) and hepatic (dual IO) | hematological, urinary, cardiovascular and hepatic | Steroid | Yes | Alive |
| 18(18) | F | 80 | Pancreatic cancer | Sintilimab | Gastrointestinal, hematological and urinary | Gastrointestinal | Steroid | No | Died of tumor progression |
| 19(19) | M | 62 | Lung cancer | Camrelizumab | Hepatic, cutaneous and endocrine | Hepatic | Steroid and mycophenolate mofetil | No | Alive |
| 20(20) | M | 48 | Clear cell renal carcinoma | Nivolumab and ipilimumab | Gastrointestinal, hepatic and urinary | Hepatic and urinary | Steroid | No | Alive |
| 21(21) | F | 57 | Clear cell renal carcinoma | Pembrolizumab | Cardiovascular, hepatic, urinary and endocrine | Cardiovascular, hepatic and urinary | Steroid | Ceftriaxone | Died of tumor progression |
| 22(22) | F | 49 | Uterine cervical cancer | Pembrolizumab | Hepatic, cardiovascular, urinary, muscular and gastrointestinal | Hepatic, cardiovascular, urinary, muscular and gastrointestinal | Steroid and tocilizumab | Cefmetazole  and  tazobactam/  piperacillin | Alive |
| 23(23) | F | 77 | Gallbladder cancer | Durvalumab | Hepatic, muscular, urinary and circulatory | Hepatic, muscular, urinary and circulatory | Steroid | No | Died |
| 24(24) | M | 57 | Oral sarcomatoid  carcinoma | Toripalimab | Hepatic and cutaneous | Hepatic and cutaneous | Steroid | No | Alive |
| 25(25) | M | 50 | Melanoma | Pembrolizumab | Nervous and endocrine | Nervous and endocrine | Steroid | Yes | Alive |
| 26(26) | M | 50 | Esophageal cancer | Tislelizumab | Hepatic, urinary and gastrointestinal | Hepatic and urinary | Steroid and mycophenolate mofetil | No | Died of tumor progression |
| 27(26) | M | 65 | Esophageal cancer | Sintilimab | Hepatic, urinary and gastrointestinal | Hepatic | Steroid | No | Alive |
| 28(26) | M | 70 | Gastric  adenocarcinoma | Tislelizumab | Hepatic, urinary, gastrointestinal and cutaneous | Hepatic and urinary | Steroid and IVIG | No | Died |
| 29(26) | M | 51 | Gastric cancer | Sintilimab | Hepatic, urinary and gastrointestinal | Hepatic | Steroid | No | Died of tumor progression |
| 30(26) | M | 65 | Lung cancer | Tislelizumab | Hepatic, urinary and gastrointestinal | Hepatic and urinary | Steroid and mycophenolate mofetil | No | Alive |
| 31(27) | M | 70 | Melanoma | Nivolumab and ipilimumab | Endocrine, gastrointestinal, urinary and nervous | Gastrointestinal | Steroid, infliximab and IVIG | No | Died of tumor progression |
| 32(28) | F | 47 | Ovarian adenocarcinoma | Toripalimab followed by tislelizumab | Endocrine and pulmonary | Pulmonary | Steroid | No | Alive |
| 33(29) | F | 70 | Lung  adenocarcinoma | Pembrolizumab | Nervous, urinary, cardiovascular, hepatic, endocrine, hematological and gastrointestinal | Nervous, urinary, cardiovascular, hepatic, endocrine, hematological and gastrointestinal | Steroid | No | Died |
| 34(30) | M | 68 | Lung squamous cell carcinoma | Tislelizumab | Endocrine, hepatic, urinary, muscular and cardiovascular | Hepatic, urinary and muscular | Steroid and IVIG | No | Alive |
| 35(31) | M | 52 | Lung squamous cell carcinoma | Pembrolizumab | Cardiovascular, musculoskeletal, pulmonary and hematological | Musculoskeletal, pulmonary and hematological | Steroid and IVIG | Imipenem cilastatin | Alive |

NA: information not available, IVIG: intravenous immunoglobulin, IO: immunotherapy

1. Pourhassan HZ, Tryon D, Schaeffer B, Mirshahidi H, Wong J. Autoimmune Rhabdomyolysis and a Multiorgan Display of Pd-1 Inhibitor Induced Immune Related Adverse Events During Treatment of Metastatic Melanoma. *Exp Hematol Oncol* (2019) 8(20):019-0140.

2. Randhawa M, Archer C, Gaughran G, Miller A, Morey A, Dua D, et al. Combined Immune Therapy Grade Iv Dermatitis in Metastatic Melanoma. *Asia Pac J Clin Oncol* (2019) 15(4):262-5.

3. Rapisuwon S, Izar B, Batenchuk C, Avila A, Mei S, Sorger P, et al. Exceptional Response and Multisystem Autoimmune-Like Toxicities Associated with the Same T Cell Clone in a Patient with Uveal Melanoma Treated with Immune Checkpoint Inhibitors. *J Immunother Cancer* (2019) 7(1):019-0533.

4. Shah N, Jacob J, Househ Z, Shiner E, Baird L, Soudy H. Unchecked Immunity: A Unique Case of Sequential Immune-Related Adverse Events with Pembrolizumab. *J Immunother Cancer* (2019) 7(1):019-0727.

5. Hyun JW, Kim GS, Kim SH, Cho JY, Kim HJ, Lee GK, et al. Fatal Simultaneous Multi-Organ Failure Following Pembrolizumab Treatment for Refractory Thymoma. *Clin Lung Cancer* (2020) 21(2):e74-e7.

6. Nowosielski M, Di Pauli F, Iglseder S, Wagner M, Hoellweger N, Nguyen VA, et al. Encephalomyeloneuritis and Arthritis after Treatment with Immune Checkpoint Inhibitors. *Neurol Neuroimmunol Neuroinflamm* (2020) 7(4).

7. Monferrer-Adsuara C, Hernández-Bel L, Hernández-Garfella ML, Remolí-Sargues L, Ortiz-Salvador M, Castro-Navarro V, et al. Case Report: Ipilimumab-Induced Multisystem Autoimmune-Like Toxicities Suggestive of Vogt-Koyanagi-Harada-Like Syndrome. *Optom Vis Sci* (2021) 98(11):1309-16.

8. Ruini C, Haas C, Mastnik S, Knott M, French LE, Schlaak M, et al. Primary Biliary Cirrhosis and Granulomatous Hepatitis after Immune Checkpoint Blockade in Patients with Metastatic Melanoma: Report of 2 Cases and Literature Discussion. *J Immunother* (2021) 44(2):71-5.

9. Sackstein P, Zaemes J, Kim C. Pembrolizumab-Induced Cytokine Release Syndrome in a Patient with Metastatic Lung Adenocarcinoma: A Case Report. *J Immunother Cancer* (2021) 9(7):2021-002855.

10. Sekai I, Hagiwara S, Watanabe T, Kudo M. A Case with Hepatic Immune-Related Adverse Events Caused by Nivolumab Exhibiting Impaired Accumulation of Regulatory T Cells. *Clin J Gastroenterol* (2021) 14(4):1191-6.

11. Xie X, Wang F, Qin Y, Lin X, Xie Z, Liu M, et al. Case Report: Fatal Multiorgan Failure and Heterochronous Pneumonitis Following Pembrolizumab Treatment in a Patient with Large-Cell Neuroendocrine Carcinoma of Lung. *Front Pharmacol* (2021) 11(569466).

12. Yang H, Zhou C, Yuan F, Guo L, Yang L, Shi Y, et al. Case Report: Severe Immune-Related Cholestatic Hepatitis and Subsequent Pneumonia after Pembrolizumab Therapy in a Geriatic Patient with Metastic Gastric Cancer. *Front Med* (2021) 8(719236).

13. Yang Y, Xu L, Wang D, Hui B, Li X, Zhou Y, et al. Anti-Pd-1 and Regorafenib Induce Severe Multisystem Adverse Events in Microsatellite Stability Metastatic Colorectal Cancer: A Case Report. *Immunotherapy* (2021) 13(16):1317-23.

14. Feng Y, Li C, Ji Y, Liu Y, Gan L, Yu Y, et al. Nivolumab Combined with Ipilimumab Treatment Induced Hypophysitis and Immune-Mediated Liver Injury in Advanced Esophageal Squamous Cell Carcinoma: A Case Report. *Front Oncol* (2022) 12(801924).

15. Hayashi H, Sawada K, Hasebe T, Nakajima S, Sawada J, Takiyama Y, et al. A Successful Case of Hepatocellular Carcinoma Treated with Atezolizumab Plus Bevacizumab with Multisystem Immune-Related Adverse Events. *Intern Med* (2022) 61(23):3497-502.

16. Yin N, Liu X, Ye X, Song W, Lu J, Chen X. Pd-1 Inhibitor Therapy Causes Multisystem Immune Adverse Reactions: A Case Report and Literature Review. *Front Oncol* (2022) 12(961266).

17. Brazel D, Lee S, Mahadevan A, Warnecke B, Parajuli R. Multiorgan Failure from Nivolumab and Ipilimumab: A Case Report and Literature Review. *Cureus* (2023) 15(7).

18. Ni CX, Zhao Y, Qian H, Fu H, Yan YY, Qiu YS, et al. Long Survival in a Pancreatic Carcinoma Patient with Multi-Organ Toxicities after Sintilimab Treatment: A Case Report. *Front Pharmacol* (2023) 14(1121122).

19. Wei T, Wang Z, Liu X. Adverse Reactions and Efficacy of Camrelizumab in Patients with Lung Adenocarcinoma with High Pd-L1 Expression: A Case Report. *Medicine* (2023) 102(7):0000000000032731.

20. Astašauskaitė S, Kupčinskaitė-Noreikienė R, Zaborienė I, Vaičiūnienė R, Vanagas T, Pranys D, et al. Multiorgan Toxicity from Dual Checkpoint Inhibitor Therapy, Resulting in a Complete Response-a Case Report. *Medicina* (2024) 60(7).

21. Di Marco A, Artioli G, Favaretto A, Cavasin N, Basso U. Multiorgan Failure Caused by Pembrolizumab and Axitinib in a Woman Affected by Metastatic Clear Cell Renal Cell Carcinoma: A Case Report and Literature Review. *Medicine* (2024) 103(13):0000000000037606.

22. Sekimata M, Kinjo Y, Tohyama A, Murakami M, Hashiwaki S, Saito Y, et al. Cytokine Release Syndrome Induced by Immune Checkpoint Inhibitor Treatment for Uterine Cervical Cancer Recurrence: A Case Report. *Oncol Lett* (2024) 28(1).

23. Anthon C, Pierret H, Houssiau F, De Cuyper A, Van Marcke C, Van Den Eynde M, et al. A Case Report of Malignant Hypertension and Multiorgan Dysfunction During Immunotherapy for Gallbladder Cancer. *Front Oncol* (2025) 15(1658621).

24. Guo W, Li Y, Li J, Dai L, Jia Y, Jiang T. Diagnosis and Treatment of a Patient with Severe Hepatitis and Rash Caused by Toripalimab: A Case Report. *Medicine* (2025) 104(35):0000000000044122.

25. Janulevicius T, Nguyen J, Briggs E. Multisystem Immune-Related Adverse Events Following Pembrolizumab: A Critical Care Case of Myocarditis, Diabetic Ketoacidosis, and Cns Demyelination. *Cureus* (2025) 17(8).

26. Jiao R, Wang C, Ying H, Nie M, Leng J, Liu Y, et al. Case Report of Immune Checkpoint Inhibitor Induced Cholestatic Hepatitis, Acute Renal Injury and Asymptomatic Pancreatic Enzyme Elevation Simultaneously. *Front Immunol* (2025) 16(1679328).

27. Jonsdottir E, Robertsson IA, Hilmarsson A, Benediktsson R, Haraldsdottir S. Multisystem Immune-Related Adverse Events During Combined Ipilimumab-Nivolumab with Subsequent Disease Progression: A Case Report. *Immunotherapy* (2025) 17:1-7.

28. Liu M, Fang S, Dai H, Li T, Guo C, Wang B. Prolonged Progression-Free Survival in a Patient with Highly Pretreated Recurrent Ovarian Cancer after Developing Multisystem Immune-Related Adverse Events: A Case Report and Literature Review. *Case Rep Oncol* (2025) 18(1):912-9.

29. Verma T, Jawadi A, Ahmed S. Management of Multiorgan Failure Caused by Immune Checkpoint Inhibitor Toxicity. *BMJ Case Rep* (2025) 18(2):2024-262209.

30. Yuan M, Han N, Shu L, Yan L, Tang H. Case Report: Multi-Organ Injuries Induced by Tislelizumab. *Front Immunol* (2025) 16(1508293).

31. Zhai M, Liu X, Li Y, Pi G, Bi J, Han G. Durable Tumor Control with Multi-Organ Immune-Related Adverse Events Following Immune Checkpoint Inhibitor and Sequential Radiotherapy in Locally Advanced Nsclc: A Case Report. *Immunotargets Ther* (2025) 14:1411-7.
